# 00001 Demographic disparities in proximity to stroke care in the United States

**DOI:** 10.1017/cts.2021.471

**Published:** 2021-03-30

**Authors:** Cathy Y. Yu, Timothy Blaine, Peter Panagos, Akash P. Kansagra

**Affiliations:** Washington University School of Medicine

## Abstract

ABSTRACT IMPACT: Given the association between lower time to treatment and better clinical outcomes in stroke patients, identifying factors correlated with reduced proximity and thus greater time to stroke care can aid efforts to reduce disparities in stroke outcomes. OBJECTIVES/GOALS: The objective of this study is to quantify the relationship between distance to the nearest certified stroke hospital and census-derived demographics of age, race/ethnicity, income, and insurance status. METHODS/STUDY POPULATION: This is a cross-sectional study. Population data for all census tracts in the contiguous United States were obtained from the US Census Bureau’s 2014-2018 American Community Survey. Stroke hospitals were identified from national or state level certification databases and were required to offer at least IV tPA. The main outcome is driving distance in kilometers from each census tract to the nearest certified stroke center, which was calculated using OSMnx, a Python package to retrieve, model and analyze real-world street networks. Quantile regression analysis was used to compare relationships between distances and tract-level demographics of age, race/ethnicity, income, and insurance status. RESULTS/ANTICIPATED RESULTS: 2,423 stroke centers and 71,929 census tracts containing 316,995,649 individuals were included. 49,918 (69%) tracts were urban. Demographic disparities in proximity to certified stroke care were greater in non-urban areas than in urban areas. Higher representation of individuals with age ≤65 years were associated with increased median distance to a certified stroke center in non-urban areas, but not urban areas. Median distance was greater with greater representation of American Indian or uninsured populations in urban and non-urban census tracts. Higher median income was associated with decreased median distance in non-urban census tracts and greater median distance in urban census tracts. DISCUSSION/SIGNIFICANCE OF FINDINGS: Reduced proximity to stroke care exists in areas with greater representation of elderly, American Indian, or uninsured persons; and low median income. These disparities are magnified in non-urban settings. Such knowledge can aid efforts to address and reduce disparities in stroke outcomes.

